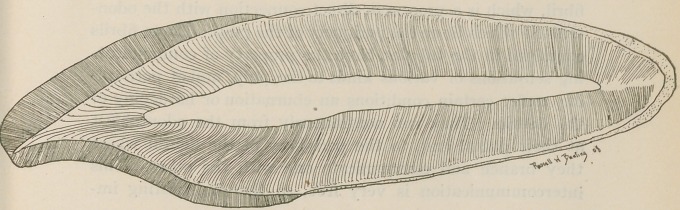# Salient Points in the Histology of the Tooth

**Published:** 1908-10-15

**Authors:** Russell Bunting

**Affiliations:** Ann Arbor, Mich.


### SALIENT POINTS IN THE HISTOLOGY OF THE TOOTH.

BY RUSSELL BUNTING, D.D.S., ANN ARBOR, MICH.

Read before the Southwestern Michigan and Fifth District Dental Societies, Grand Rapids, Mich., April 14 and 15, 1908.

Just as the surgeon must know the minute anatomy of the parts upon which he works, the exact position and arrangement of the tissues beneath his knife, so must the dentist be thoroughly acquainted with the structural arrangement and position of the constituents of the tooth, to which he confines so much of his attention, and upon which he performs so large a part of his operations. Every operation which a dentist makes upon a tooth is more or less dependent upon the histological structure of the part involved, and whether he takes these into consideration or not, they determine, to a large extent, the success or failure of the operation. It is very proper, therefore, to review the special anatomy and histology of the tooth from time to time, and we may apply this knowledge to the specific methods which we employ in the treatment of the teeth, in order that our operations may be scientifically correct, and truly deserving of the claim that "Dentistry is an exact science."

Along this line I wish to present for your consideration a pair of charts representing an enlargement of an incisor and a molar, with the aid of which I will call your attention to the salient points in the histology of the teeth represented, and their bearing upon a few of our dental operations.

The enamel is a protective casement for that portion of the tooth which projects above the gum. It is composed of rods of calcific material joined together by thin layers of cement substance and so arranged as to give the greatest possible resistance to strain applied in the force of mastication. In general, the rods run radially from the dentin to the surface, so that any strain applied will be upon the ends of the enamel rods and in a direction parallel to their course. In the cusps of the molars and bicuspids, where the bulk of the strain in mastication is applied, the rods are very much twisted and interwoven, making them almost incapable of cleavage. As long as the enamel remains intact it is very resistent to forces of every kind, but, as pointed out by Dr. Noyes, when once a break has been made in its continuity and we attempt to repair that break, we must so prepare the edges of the enamel which border on the cavity that all exposed rods shall run uninterruptedly to the dentin. For that reason all cavities occurring upon the occlusal surface of the molars and bicuspids may be prepared with a very slight bevel to the enamel margins, inasmuch as the rods tend to run from the dentin toward the cavity, while in the case of cavities formed on the lateral surfaces of the teeth much more bevel must be made, as the rods tend to run away from the cavity.

In the preparation of all cavities the direction of the enamel rods must be taken into account. Something of their course may be ascertained by cleaving the enamel margin with a chisel, as the enamel will cleave more readily along the lines of cement which bind the rods together than in any other direction, if the rods are straight. In teeth which develop from a single center—as the incisors and cuspids—the direction of the rods, is in general, perpendicular to the surface of the tooth. However, in the teeth which have two or more centers of calcification—as in the molars and bicuspid—the rods on the occlusal surface radiate from these centers. The areas in which the enamel from two or more centers unite are poorly calcified, and a cleft is often left forming what is perhaps the most vulnerable point in the tooth to the action of decay. As we have said, repair of this class of cavity is favored by the direction of the enamel rods, but if the cavity is extensive enough to encroach upon the centers of calcification, great care must be used in preparation of the enamel walls on account of the fact that at this point the enamel rods are very gnarled and twisted, especially near the dentin, and it is therefore difficult to obtain rods for margins which run uninterruptedly to the dentin.

On the lateral surfaces of the tooth the enamel rods in the middle third run nearly horizontal with a slightly occlusal inclination, especially near the junction of the occlusal and middle thirds. But in gingival third it is seen that the direction is markedly inclined toward the cervix, especially near the cementum-enamel junction. For this reason the gingival borders of all cavities which extend into this region, must have their enamel margins sloped away far enough to get beyond the plane of the rods. Furthermore, when approximal cavities extend very near to the cementum-enamel junction there will be left a triangular strip of enamel, composed of short rods, which will form the cervical margin of the cavity; this margin, because of its frailty and non supports, will be very likely to be chipped off under the malleting, or so weakened that it will fall out of position at some later time in the life of the filling. It is not improbable that the failure of many of our approximal fillings at the cervical border may be due to the faulty preparation of the enamel margins at this point, rather than to any fault in the adaptation of the filling material.

The enamel when fully formed, remains throughout life in the same density and degree of hardness, except as it may be weakened or disintegrated by a destructive force—in other words, the enamel has no means of throwing up a defense against an invading force, or of recuperating after the cessation of an injury. Injuries to the enamel are of three classes: (1) Wear or abrasion. (2) Fractures. (3) Acid decalcification.

In the case of wear the enamel is wasted evenly and to an extent determined by the character of the antagonizing force. Fractures extend along the lines of cement beween the rods and are determined in their course by the nature of the force applied. Decalcification of the enamel is of two kinds—erosion and decay. In each there is a solution, first of the cement substance between the rods and then of the rods themselves. As the cement substance is the most easily attacked, the decalcifications run much more readily toward the dentin, between the rods which are affected, rather than in a lateral direction; this is the reason why we find so many cavities of considerable extent in the dentin, which have but a pin-pcint aperture through the enamel.

The dentine is composed of a ground substance, through which run the dentinal tubules. The ground substance is of a calcific nature, but with a considerable amount of organic substance included in its formation, The tubules of the dentin are so numerous that they ccmprise something more than a tenth of the total bulk of the dentin. These tubules take their course, uninterruptedly, from the pulp chamber to the enamel in the coronal portion of the tooth, and from the pulp canals to the cementum in the root portion. Their course in the coronal portion is along two definite curves, so called "primary" and "secondary" curves, and the tubules in this region are the longest and most tortuous of any part of the tooth. Toward the middle of the tooth the tubules are less curved and take a nearly straight course from the pulp to the periphery of the dentin. In the root portion they run in very nearly straight lines, except at the apex, where they are seen to radiate.

In the injection of cocain into the pulp by means of a high pressure syringe, it is necessary to bear in mind the exact direction of the tubules in the dentin to which the syringe is applied, inasmuch as better results will be obtained by bringing the point in direct contact with the ends of a few tubules, than at any angle to them. It will be noticed that in the anterior teeth any contact made upon the dentin will be upon tubules which angle off sharply toward the gingival, this angle being more pronounced toward the incisal end and less so toward the cervix. In the crowns of the molars and bicuspids the central tubules take a nearly straight course to the pulp, parallel to the long axis of the tooth, those under the cusps angle toward the center of the tooth with little curve, while those on the lateral surfaces of the tooth have the primary and secondary curves similar to those in the anterior teeth. It will be seen from the drawings that in many teeth it would be more advantageous to make the contact point for a high pressure syringe at or near the convex of the tooth than further up in the crown, for the reasons that the tubules are often shorter and straighter in this region and that solutions forced into the pulp from the cervex enter it at a much lower level, which latter is a decided advantage in cases where the coronal portion of the pulp is diseased, or may have formed a layer of secondary dentine over itself.

The dentinal tubules are not empty, but contain, as long as the pulp is living and healthy, a living protoplasmic fibril, which is a process in direct connection with the odontoblasts which lie on the periphery of the pulp. These fibrils contained in the tubules, have the property of communicating sensations of various kinds to the pulp, and of performing under certain conditions an eburnation or hardening of the dentin. They run continuously from the odontoblast at their pulpal end, to the periphery of the dentin where they branch and communicate one with the other. This intercommunication is very free, so that an irritating impulse applied at the junction of the dentin and the enamel is carried to the pulp not only by the fibrils which are immediately affected, but is also transferred to neighboring fibrils, in a manner like to messages sent out over wires running from a central telephonic switchboard. It is because of this very free communication that we often find that cavities are far more sensitive to the bur or excavator at the dento-enamel junction than at the bottom of the cavity, even though the decay is very much nearer to the pulp.

The process of eburnation of the dentin is accomplished in the dentinal fibrils by their deposition of lime salts in the tubules in which they lie and in their own substance, and when the lime salts become calcified the dentin contains in that area no tubules, but simply a solid homogeneous mass of calcific material. An area in the dentin which has undergone such a change as this is spoken of as the "Transparent Zone," and is produced in living teeth only, as a barricade against invading caries which threatens the life of the pulp and the destruction of the tooth. This hardening process takes place only in those tubules which are directly affected by the injury, and is in the form of a cone shaped area, with the base toward the enamel and the tapering to the pulp.

In the case of abrasion, when the enamel has been worn away, if a transparent zone has been formed in the dentin below, a homogeneous surface of dentin without any tubules will be presented to the invading action. This change must modify the rate of advance of the abrasion to a marked degree,

Acid decalcifications, when they reach the dentin, undoubtedly travel much more rapidly along the lumen of the tubules than in the substance between them, so that if the tubules become filled up with a material similar to the intertubular substance, the progress of the acid is seriously impeded. It is hard to estimate the protective influence which this calcification exerts in the tooth, especially in the cases of erosions and decays.

When caries has gained access through the enamel to the dentin, it progresses toward the pulp along the line of the tubules, even if there has been a calcification of the canals. There is also a considerable lateral spread of the carious process between the dentin and the enamel at their junction, which is due to the poor calcification of this region. In the lateral spread, caries may effect the enamel by undermining it, causing it to be weakened, or by initiating recurrent caries of the enamel. On the other hand, in the dentin, it may carry the infection to tubules lying outside of the affected zone, thus increasing the area of decay. It is in this dento-enamel junction, or interzonal layer, that many cases of recurrent caries, which have their infection coming in from the border of the filling, get their first foothold in the tooth. It is unfortunate that this very vulnerable part of the tooth should be so sensitive, for it is very likely that a good percentage of cases of recurrent caries are due to an incomplete removal of the decayed tooth substance just beneath the enamel, either from the extreme sensitivity of that part, or to a lack of care in the cavity preparation.

As the line of decay follows the course of the tubules, in some instances the carious process will narrow down to a small zone as it approaches the pulp. So that in the preparation of some cavities which penetrate into the dentin a considerable distance, we may have a solid cavity floor, in which there is but a small spot of decay continuing still deeper, and this spot of infection escaping the eye of the operator and being left under the filling, may give rise to a recurrence of the carious process.

In conclusion let me repeat the points of histological interest to be noted in the preparation of a cavity for filling. In the enamel, sound walls with margins composed of parallel rods which run continuously to the dentin; in the dentin, a complete removal of all affected tooth substance, especial care being taken at the dento-enamel junction and in the base of the cavity in the direction which the main advance of the carious process entered and extended.

### DISCUSSION.

Dr. E. B. Spalding: One or two points occur to me in listening to Dr. Bunting's very excellent paper; and one is that if we could have a better understanding of our histology we should often explain some of our failures. For instance when we find a frail edge broken down, we don't stop to discover that there is a reason for that. We think we probably were not accountable for that, but if we would bear in mind the direct form of the enamel rods, the direction in which they run and especially in the cusps of the teeth, which I am sure I don't think of frequently enough, radiating from the dentin as they do. I am sure this paper will make me more mindful in my cavity preparation hereafter. Another thing, in the use of the high pressure syringe, we sometimes get rather warm under the collar when we cannot make it work, and he has explained it very satisfactorily by the deposition of the secondary dentin. It would seem as though it was desirable on many occasions to use something to desensitize, whereby we can more completely prepare the cavity. There can be little doubt in our minds that the infected area immediately under the enamel has been completely removed, and that all decay has been completely removed from every portion of the cavity, and that it is better even to make our opening for a high pressure syringe elsewhere. If we can make a separate cavity at the portion next to the gum line where Dr. Bunting has shown us, results would be very much more effective on account of the course of the tubulae.

Dr. Bunting: I am sorry that I have to do all the talking. I was in hopes that I could get some of the other members to say something upon this work. This is my line at college so that I could talk all the rest of the morning, but I presume I have said all that is necessary at this time. I only hope that some of you may get to thinking upon these things as I think of them all the while, for they help me in preparing cavities. I hoped this paper would stir up some of you to thinking more about the histology of the tooth. What I wanted to know more especially was whether or not some of you have noticed some of these things I have brought out in reference to the injection of cocain; or whether or not you can get much better results through the lower part of the tooth; whether or not you have seen cases that you felt the gingival borders of your cavities were dropping out rather than decaying. I am also wondering whether experience will bear out these theories, or is it all hot air that I have been talking all this time?

DR. E. T. LOEFFLER: The point that the doctor speaks of in regard to the use of the injection of cocain by means of a high-pressure syringe: I can say this much, that either at the cervical margin or in the direction of the axis of the tooth are altogether the best points to use. Very frequently using the cocain with high-pressure syringe along the axis of the tooth gives altogether the best results. It works fully as well, or in some cases, I think, perhaps better as you get results quicker than by using the cervical margin, although, as he stated, that means an extra cavity, an extra pit to fill, which is undesirable. In many cases the high pressure syringe is used to remove the pulp, but we can, of course, remove the pulp to much better advantage by having an opening which will go directly into the pulp cavity. So that in a great majority of cases it is much better to make that opening along the axis of the tooth. Those of us who have had experience with high-pressure know that the point of contact means everything. The cup-shaped surface, to begin with, is healthy enamel, this bit extending in the direction of the tubuli. There is one point, of course, where we have a good deal to contend with, that is one phase of this secondary dentin, but even in such cases I frequently succeed by having a good point of contact.

Now this paper in that sense is extremely interesting; it is in all phases that he spoke of, but particularly in this work of using cocain by means of the high pressure- syringe.

To understand, or to review at times the histological structure of the tooth, should make it easier to do this work more readily, and we can often succeed where we have had failures before. Students, I find, time and again will fail in an operation, simply because they did not bear in mind their histology which they had a year or two before.

## Figures and Tables

**Figure f1:**
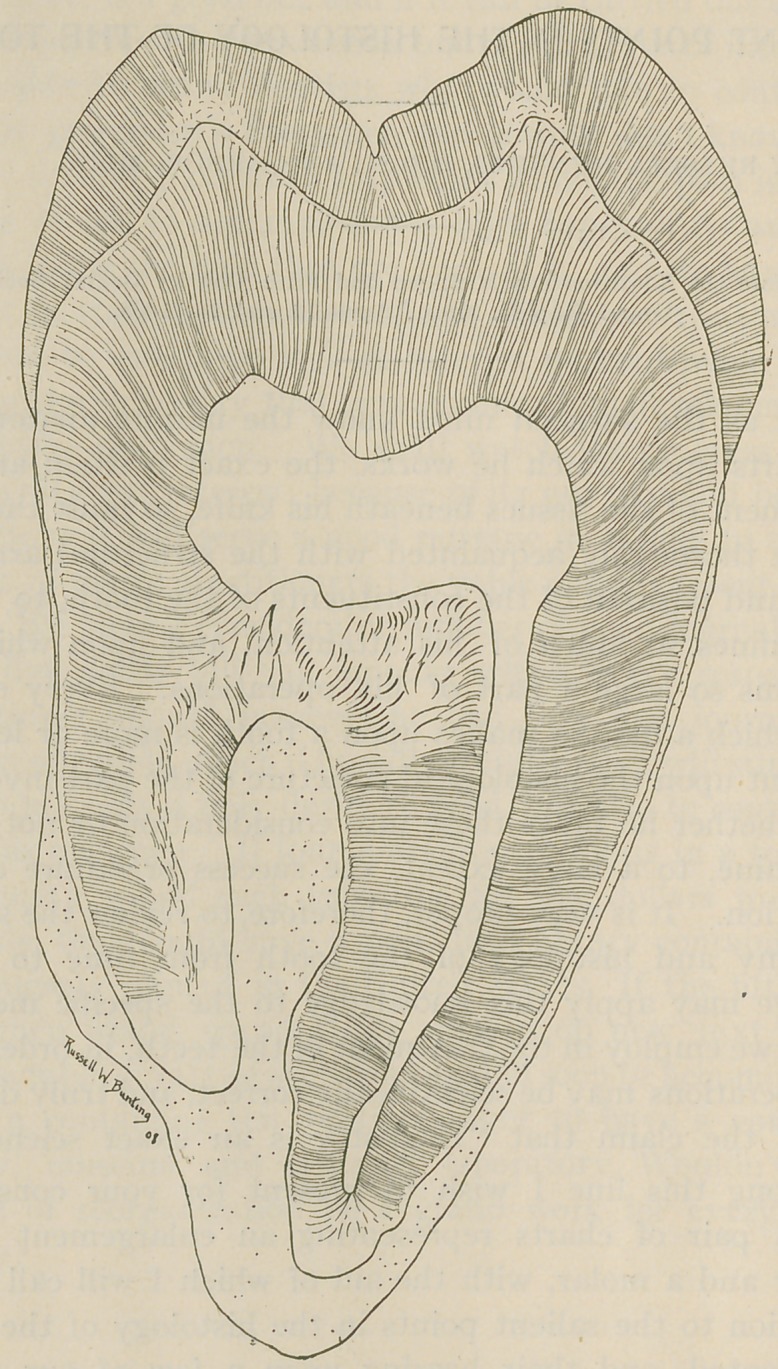


**Figure f2:**